# Elevated platelet count is associated with decreased mortality from hemorrhagic stroke in hospital: a multi-center retrospective cohort study

**DOI:** 10.1038/s41598-024-53956-7

**Published:** 2024-02-15

**Authors:** Zhenhua Huang, Chenglin Liu, Zhanxing Wu, Xiaoyong Xiao, Zhongqin Chen, Qun Huang, Dehong Liu, Zhe Deng

**Affiliations:** 1 Department of Emergency, Shenzhen Second People’s Hospital and The First Affiliated Hospital of Shenzhen University, Shenzhen, 518035 China; 2 Shenzhen Second People’s Hospital and The First Affiliated Hospital of Shenzhen University, Guangzhou Medical University, Shenzhen, 518035 China

**Keywords:** Platelet count, Hemorrhagic stroke, Mortality, Non-linear, Association, Prognostic markers, Stroke

## Abstract

This study aimed to investigate the relationship between platelet count (PC) and mortality in patients with hemorrhagic stroke (HS). The research reviewed data from 10,466 patients hospitalized in 208 hospitals in the United States from January 1, 2014, to December 31, 2015. Of these, 3262 HS patients were included in the primary analysis for those admitted to the intensive care unit (ICU). The average age of these patients was 67.05 years, with 52.79% being male. The median PC was (221.67 ± 73.78) × 10^9^/L. Multivariate logistic regression analysis revealed that PC was a protective factor for mortality in HS patients (OR = 0.98, 95% CI 0.97–1.00, *P* < 0.05). Additionally, a non-linear association between PC and mortality in HS patients was found using a generalized additive model (GAM) and smooth curve fitting (penalty spline method). For the first time, a recursive algorithm identified the inflection point of platelet count as 194 × 10^9^/L. On the left side of the inflection point, for every increase of 10 units in platelet count, the mortality rate of HS patients decreases by 10%. The study demonstrates a non-linear relationship between PC and the risk of mortality in HS patients. A platelet counts higher than the inflection point (194 × 10^9^/L) may be a significant intervention to reduce mortality in HS patients.

## Introduction

Hemorrhagic stroke (HS) is among the most common subtypes of stroke and is associated with high mortality and severe disability^1^. Asia has a higher rate of HS compared to the United States and Europe, with the incidence in China being as high as 23.8%^2^. Among all stroke subtypes, hemorrhagic stroke has the highest mortality rate and profound effects on individuals and society^3^. Currently, there are various treatment options for HS, including surgery, control of intracranial hypertension and blood pressure, and rehabilitation. However, there remains controversy surrounding the optimal method of treatment for HS^4^. Hemostasis plays a key role in preventing the progression of hematoma in HS. Previous studies showed that platelets, small anucleated blood cells, play an important role in hemostasis and thrombosis^5–7^. It was also reported that platelet levels might affect the morbidity and mortality of patients with cardiovascular disease^8,9^. Thrombocytopenia was shown to have adverse effects on patients admitted to the intensive care unit (ICU)^10,11^. However, the role of platelets in HS remains unclear.

Recently, research on the association between platelet and HS attracted substantial attention. Accumulating evidence suggested that platelet dysfunction was associated with the mortality of HS patients^12,13^. Du et al.^12^ reported that an elevated platelet count (PC) was associated with a decreased risk of HS, while Mayda-Domaç et al.^13^ found that lower PC levels were associated with HS. Guidelines recommend that HS patients with thrombocytopenia should receive platelet concentrate^14^. Despite numerous research in this field, the prognostic value of PC in hospitalized HS patients remains to be clarified.

The transfusing of a minimum platelet threshold of 50 × 10^9^ /L for trauma, preprocedural and major bleeding is widely upheld^15–17^, but without good-quality evidence. Currently, the best threshold for patients who received platelet transfusions remains unclear, and in some cases, this depends on the doctor’s clinical experience and the patient’s clinical diagnosis^18^. This retrospective study aimed to explore the association between PC and mortality in hospitalized stroke patients, and to identify the optimal PC threshold for minimizing mortality in HS patients.

## Results

### Baseline characteristics

Table 1 summarizes the demographic and clinical characteristics of the 3,262 hemorrhagic stroke patients included in the study, with a mean age of 67.05 ± 14.89 years. Among them, 52.79% were male, and 47.21% were female, predominantly Caucasian (74.92%). Throughout the study, 13.67% experienced in-hospital mortality. The median platelet count was 221.67 ± 73.78 × 10^9/L, mostly within the normal range.

**Table 1:** The baseline characteristics of participants.

Platelet Count (× 10^9^/L)	T1(< 100) (*n* = 121)	T2(100–300) (*n* = 2674)	T3(> 300) (*n* = 467)	*P*-value
age(year)	63.27 ± 13.49	67.88 ± 14.56	63.30 ± 16.30	< 0.001
BMI (kg/m 2 )	28.01 ± 8.26	27.73 ± 8.56	28.08 ± 9.76	0.701
Sex				< 0.001
Male, *n* (%)	74 (61.16)	1448 (54.15)	200 (42.83)	
Female, *n* (%)	47 (38.84)	1226 (45.85)	267 (57.17)	
Ethnicity				0.199
African-American, *n* (%)	19 (15.79)	313 (11.74)	74 (15.91)	
Asian, *n* (%)	1 (0.84)	57 (2.14)	5 (1.08)	
Caucasian, *n* (%)	84 (70.59)	2008 (75.35)	342 (73.55)	
Hispanic, *n* (%)	9 (7.56)	127 (4.77)	20 (4.30)	
Native American, *n* (%)	0 (0.00)	11 (0.41)	2 (0.43)	
Unknown, *n* (%)	6 (5.04)	149 (5.59)	22 (4.73)	
Scr (mg/dl)	1.41 ± 0.95	1.22 ± 1.10	1.16 ± 0.89	0.063
BUN (mmol/L)	28.89 ± 20.98	21.13 ± 14.28	21.52 ± 18.44	< 0.001
TC (mmol/L)	125.20 ± 48.25	161.13 ± 46.87	165.79 ± 57.60	< 0.001
TG (mmol/L)	126.53 ± 70.47	127.86 ± 95.19	137.90 ± 114.05	0.281
RBC (× 10^12^/L)	3.66 ± 0.80	4.31 ± 0.72	4.24 ± 0.78	< 0.001
HGB (g/L)	11.38 ± 2.46	12.95 ± 2.20	12.30 ± 2.35	< 0.001
History				
ACS, *n* (%)	6 (4.96)	111 (4.15)	23 (4.93)	0.699
AF, *n* (%)	18 (14.88)	340 (12.72)	48 (10.28)	0.241
CHF, *n* (%)	11 (9.09)	137 (5.12)	19 (4.07)	0.082
Coagulopathy, *n* (%)	9 (7.44)	43 (1.61)	9 (1.93)	0.883
Diabetes, *n* (%)	14 (11.57)	333 (12.46)	55 (11.78)	0.890
Hypertension, *n* (%)	25 (20.66)	840 (31.41)	151 (32.33)	0.037
Cancer, *n* (%)	4 (3.31)	16 (0.60)	2 (0.43)	0.001
sepsis, *n* (%)	26 (21.49)	121 (4.53)	28 (6.00)	0.030
Antiplatelet, *n* (%)	7 (5.79)	136 (5.09)	25 (5.35)	0.922
Anticoagulant, *n* (%)	2 (1.65)	43 (1.61)	9 (1.93)	0.883
Cephalosporin, *n* (%)	7 (5.97)	88 (3.29)	21 (4.05)	0.173
Hospital mortality				< 0.001
No, *n* (%)	86 (71.01)	2314 (86.54)	448 (95.53)	
Yes, *n* (%)	35 (28.93)	360(13.46)	51 (10.92)	

Continuous variables are summarized as mean (SD) or median (quartile interval); categorical variables are presented as percentages (%).

BMI, body mass index, TG, triglyceride; TC, total cholesterol; BUN, blood urea nitrogen; Scr, serum creatinine; PC, Platelet count; RBC, read blood cell; HGB, hemoglobin; ACS, acute coronary syndrome; AF, atrial fibrillation; CHF, congestive heart-failure.

Participants were categorized into three platelet groups: T1 (< 100 × 10^9^/L), T2 (100–300 × 10^9^/L), and T3 (> 300 × 10^9^/L). In comparison to T1, T2 and T3 showed significant increases in age, female gender, total cholesterol (TC), red blood cells (RBC), and hemoglobin (HGB), as well as hypertension compared to the low platelet group (T1: < 100). Conversely, the male gender, blood urea nitrogen (BUN) levels, cancer rate, sepsis rate, and hospital mortality rate decreased.

### Factors influencing the risk of hospitalized HS patients’ death by univariate analysis

As shown in Table 2, univariate analyses showed that PC was associated with the hospital mortality of HS patients (*P* < 0.05). Similar results were found for age, levels of HGB, BUN, ACS rate, CHF rate, diabetes rate and sepsis rates (all *P* < 0.05). However, hospitalized HS patients’ death was not associated with sex, ethnicity, BMI, cancer rate, hypertension rate, GIB rate, or drugs used (all *P* > 0.05).

**Table 2:** Factors influencing risk of hospitalized HS patients’ death analyzed by univariate analysis.

	Statistics	OR (95% CI)	*P*-value
Sex			
Male, *n* (%)	1722 (52.79)	1.0	
Female, *n* (%)	1540 (47.21)	0.95 (0.78, 1.17)	0.6417
Age (years)	67.05 ± 14.89	1.01 (1.01, 1.02)	< 0.0001
Ethnicity			
African-American, *n* (%)	406 (12.50)	1.0	
Asian, *n* (%)	63 (1.94)	1.06 (0.50, 2.28)	0.8737
Caucasian, *n* (%)	2434 (74.92)	0.99 (0.73, 1.35)	0.9691
Hispanic, *n* (%)	156 (4.80)	1.05 (0.61, 1.79)	0.8638
Native American, *n* (%)	13 (0.40)	0.00 (0.00, inf.)	0.9586
Unknown, *n* (%)	177 (5.45)	1.25 (0.77, 2.04)	0.3704
BMI (kg/m ^2^)	27.79 ± 8.73	1.00 (0.99, 1.01)	0.7202
HBG (g/L)	12.80 ± 2.26	0.93 (0.89, 0.97)	0.0016
BUN (mmol/L)	21.47 ± 15.30	1.02 (1.01, 1.02)	< 0.0001
PC (× 10^9^/L)	221.67 ± 73.78	0.98 (0.97, 0.99)	0.0028
RBC (× 10^12^/L)	4.27 ± 0.74	0.81 (0.71, 0.93)	0.0500
ACS, *n* (%)	140 (4.29	2.02 (1.35, 3.03)	0.0006
CHF, *n* (%)	167 (5.12)	1.66 (1.13, 2.46)	0.0106
COPD, *n* (%)	119 (3.65)	1.29 (0.79, 2.11)	0.3119
Diabetes, *n* (%)	402 (12.32)	1.63 (1.24, 2.14)	0.0004
Cancer, *n* (%)	22 (0.67)	1.41 (0.47, 4.18)	0.5387
Hypertension, *n* (%)	1016 (31.15)	1.11 (0.90, 1.38)	0.3175
GIB, *n* (%)	21 (0.64)	0.32 (0.04, 2.36)	0.2606
Sepsis, *n* (%)	175 (5.36)	2.47 (1.74, 3.51)	< 0.0001
Antiplatelet, *n* (%)	168 (5.15)	1.11 (0.72, 1.72)	0.6399
Anticoagulant, *n* (%)	54 (1.66)	0.94 (0.42, 2.09)	0.8784
Use cephalosporin	116 (3.56)	1.33 (0.81, 2.18)	0.2561

### Relationship between PC and mortality of HS patients in different models

In the unadjusted model (crude model), a 10-unit increase in PC was associated with a 20% decrease in the risk of HS patients’ death in the hospital (HR = 0.98, 95% CI: 0.97–0.99 *P* = 0.0028). In the minimally adjusted model (model I), each additional 10-unit increase in PC decreased the risk of death of hospitalized HS patients by 20% (OR = 0.98, 95% CI 0.97–1.00, *P* = 0.00159). In the fully adjusted model (model II), each additional 10-unit increase in PC was accompanied by a 20% decrease in death risk in HS patients in the hospital (OR = 0.98, 95% CI 0.97–1.00, *P* = 0.00259). The distribution of the confidence intervals indicated that the relationship between PC and death risk in HS patients was reliable and stable. However, when we assessed the subgroups based on PC, the results showed that the hospital mortality of HS patients was lower in T2 and T3 compared with T1 (Table 3).

**Table 3:** Relationship between PC and hospitalized HS patients’ death in different models.

Exposure	Non-adjusted model (OR,95%, *P*)	Adjusted model I (OR,95%, *P*)	Adjusted model II (OR,95%, *P*)
PC (× 10^9^/L)	0.98 (0.97, 0.99) 0.0028	0.98 (0.97, 1.00) 0.0159	0.98 (0.97, 1.00) 0.0259
PC (Trisection)			
T1	Ref	Ref	Ref
T2	0.38 (0.25, 0.58) < 0.0001	0.36 (0.24, 0.55) < 0.0001	0.42 (0.27, 0.64) < 0.0001
T3	0.30 (0.18, 0.49) < 0.0001	0.30 (0.18, 0.50) < 0.0001	0.34 (0.20, 0.56) < 0.0001
*P* for trend	0.0001	0.0003	0.0008
N	3262	3249	3249

Non-adjusted model: no covariates were adjusted for.

Adjusted model I: we only adjusted for age, sex and ethnicity.

Adjusted model II: we adjusted for age, sex, ethnicity, AF, ACS, diabetes and sepsis.

### Non-linear relationship of PC with in-hospital mortality of HS patients

**Figure 1:**
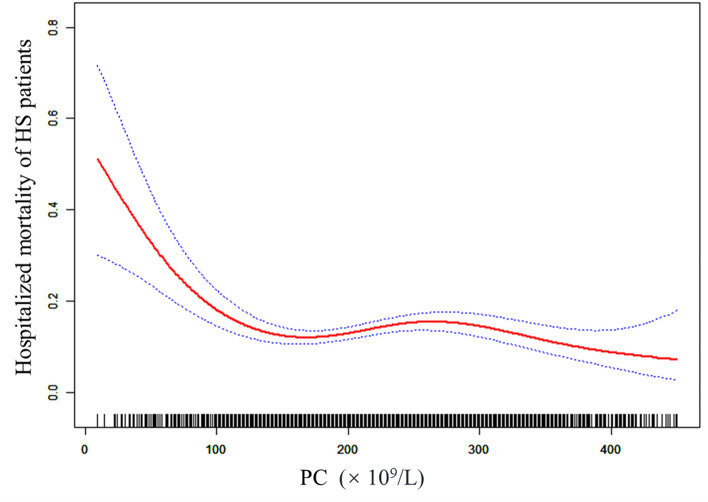
The nonlinear relationship of PC with in-hospital mortality of HS patients. The smoothing splines were generated utilizing generalized additive model and adjusted for age, sex, ethnicity, AF, ACS, diabetes and sepsis. The red line indicates the risk of mortality and the blue dot line indicates 95% confidence intervals.

Smoothing splines were generated utilizing a generalized additive model and adjusted for age, sex, ethnicity, AF, ACS, diabetes and sepsis. The results showed that the link between PC and mortality of HS patients was non-linear (Fig. 1). In addition, we found that the inflection point of PC was 194 × 10^9^/L using a recursive algorithm. Then, we calculated the OR and CI on the left and right of the inflection point. The results showed that the HR was 0.99 on the left side of the inflection point (95% CI: 0.99–1.00) (*P* = 0.0026). In addition, when PC was < 194 × 10^9^/L, a 10-unit (× 10^9^/L) increase in PC was associated with a 10% decrease in HS-related morality. The HR was 1.00 on the right side of the inflection point (95% CI:1.00–1.00) (*P* = 0.8417), but the difference was not statistically significant (Table 4).

**Table4:** The result of the two-piecewise linear regression model.

hemorrhagic stroke patients	OR, 95%CI	*P*
Inflection points of PC (10^9^/L)	194	
≤ 194	0.99 (0.99, 1.00)	0.0026
> 194	1.00 (1.00, 1.00)	0.8417
*P* for log-likelihood ratio test	0.026	

## Discussion

This retrospective cohort study examined the association between PC and the mortality of HS patients and demonstrated that PC was an independent risk factor for the mortality of hospitalized HS patients. Moreover, the present study observed a non-linear relationship between PC and the risk of in-hospital death in HS patients. In addition, we first demonstrated that the inflection point of PC was 194 × 10^9^/L, and notably, when PC was < 194 × 10^9^/L, the magnitude of PC increase correlated significantly with the decrease in all-cause mortality in HS patients.

HS occurs as a result of cerebral hemorrhage due to the rupture of blood vessels, which can lead to severe morbidity and high mortality. Progression of HS has been associated with worse patient prognoses and severity of HS. The risk factors for mortality in hemorrhagic stroke primarily involve hypertension, impaired erythrocyte deformability, and endothelial dysfunction. Each of these factors may contribute to an increased risk of bleeding^19,20^. Currently, some studies reported PC as a good predictor of mortality in HS. Platelet dysfunction, for instance, based on vascular endothelial growth factors released from activated platelets, was reported to be a crucial factor inducing HS^21,22^. Therefore, it is essential to actively search for the association between platelet and the prognosis of HS patients to identify ways to improve their treatment outcomes.

A retrospective study involving 445 cases found that an increased platelet count is a risk factor for ischemic stroke, while a decreased PC is a risk factor for hemorrhagic stroke^12^. Another prospective study involving 738 patients with acute ischemic/hemorrhagic stroke, including 44 with ischemic stroke and 29 with hemorrhagic stroke, found a negative correlation between platelet count and hemorrhagic stroke. However, this study was a single-center study with a small sample size, possibly leading to selection bias^13^. A retrospective study using prospectively collected data found that the platelet-to-lymphocyte ratio (PLR) was significantly associated with poor prognosis and mortality risk in patients before and 24 h after rtPA thrombolytic therapy^23^. However, there are currently no studies on the relationship between platelet count and the prognosis of hemorrhagic stroke. Therefore, this study explored the correlation between platelet count and in-hospital mortality in hemorrhagic stroke.

In this study, a cohort of 3262 HS patients was examined. Upon admission, the platelet levels of most patients fell within the normal range, with only a few presenting thrombocytopenia, a common marker in critically ill individuals. Notably, our analysis revealed a significantly higher mortality rate among HS patients in the low PC group compared to the high PC group. Furthermore, a negative correlation was observed between increasing PC levels and a decrease in in-hospital mortality among HS patients. Employing GAM and smooth curve fitting, our investigation highlighted a non-linear relationship between PC levels and the risk of in-hospital mortality in HS patients. After adjusting for confounding factors, the inflection point of PC was determined to be 194 × 10^9^/L. Below this threshold, a 10-unit increase in PC correlated with a noteworthy 10% reduction in in-hospital mortality for HS patients. Intriguingly, when PC exceeded 194 × 10^9^/L, the risk of hospital death in HS did not exhibit a significant decrease with further increases in PC. These findings emphasize that the elevation of PC, if it remains below 194 × 10^9^/L, serves as an independent protective factor for patients with HS. The outcomes of our study address a longstanding issue and furnish a solid clinical foundation for the treatment of hemorrhagic strokes.

Compared to other studies, our research is the first to explore the association between platelet count and the prognosis of patients with hemorrhagic stroke. In addition, the robustness of the data is related to the relatively large sample size, based on which we observed a non-linear relationship between PC and the risk of in-hospital death in HS patients. Further, we also found that the inflection point of PC was 194 × 10^9^/L, which played a crucial role in modulating platelet to improve the prognosis of HS in clinical practice.

The primary reasons for thrombocytopenia in HS patients include increased platelet destruction, reduced platelet production, platelet dilution, or aggregation. For instance, patients with immune diseases like systemic lupus erythematosus are most prone to thrombocytopenia^24^. Moreover, patients without systemic diseases might also have thrombocytopenia, potentially induced by pharmacological agents like antiplatelet drugs and cephalosporins^25,26^. Additionally, HS complicated by severe sepsis and hypersplenism can directly diminish platelet count. Furthermore, other severe illnesses might impair the bone marrow’s hematopoietic function, leading to diminished platelet levels^27–29^. Based on this study’s findings, appropriate etiological treatment or platelet transfusion may be beneficial for the prognosis of HS patients.

The results of this study have certain clinical implications. Firstly, the study highlighted that in patients with HS, a low PC is significantly associated with higher in-hospital mortality. This emphasizes the importance of monitoring patients’ platelet count in the early assessment of hemorrhagic stroke. Secondly, by using GAM and smoothing curve fitting, the study identified the inflection point of PC levels as 194 × 10^9^/L. This finding suggests that for each increase of 10 count units below this threshold, there is a significant reduction in in-hospital mortality for HS patients. Therefore, the PC level becomes a clinical indicator for predicting the in-hospital survival rate of patients. The results underline that when PC levels rise but remain below 194 × 10^9^/L, they act as an independent protective factor, significantly reducing the risk of in-hospital mortality in HS patients. This provides a guideline for clinicians, suggesting that maintaining or increasing platelet levels within this range may be beneficial for patients’ prognosis.

However, there were also some potential limitations associated with this study. First, this was a retrospective analysis of a prospective registry study. We did not have data on multiple PC at different time intervals; hence, we could not explore the association between the change in PC with HS patients’ prognoses. Further, we could not determine long-term outcomes because only short-term follow-up data were available in the database. Thus, further studies, such as randomized clinical trials, on the relationship between PC and stroke are needed to confirm our findings.

## Conclusion

For the first time, we report a non-linear relationship between PC and mortality risk in hospitalized HS patients. An elevated PC above the inflection point (194 × 10^9^/L) could lead to a decreased mortality of HS patients. when PC was > 194 × 10^9^/L, the risk of in-hospital death in HS did not significantly decrease with an increase in the PC.

## Methods

### Ethics statement

This study analyzed a publicly available, anonymized database with the Institutional Review Board of the Massachusetts Institute of Technology (Cambridge, Massachusetts, USA) approval, which permits unrestricted use, distribution, and reproduction in any medium. One author (Zhenhua Huang) obtained access rights and was responsible for data extraction (certification number:49995491). The data was the current secondary analysis, which has not required ethical approval. All methods were carried out in accordance with relevant guidelines and regulations.

### Data source

The data analyzed in this study were extracted from the eICU-CRD, a multicenter intensive care unit database containing over 200,000 cases^30,31^ As a multicenter database, the e-ICU platform documents the electronic medical record of patients from 208 hospitals in the United States from 2014 to 2015. All data were extracted using Structured Query Language (SQL) before further analysis. The data of interest to this present study included gender, age, ethnicity, BMI, atrial fibrillation (AF), congestive heart failure (CHF), acute coronary syndrome (ACS), hypertension, diabetes mellitus, chronic obstructive pulmonary disease (COPD), biochemical criterion and drugs used.

### Study population

**Figure 2:**
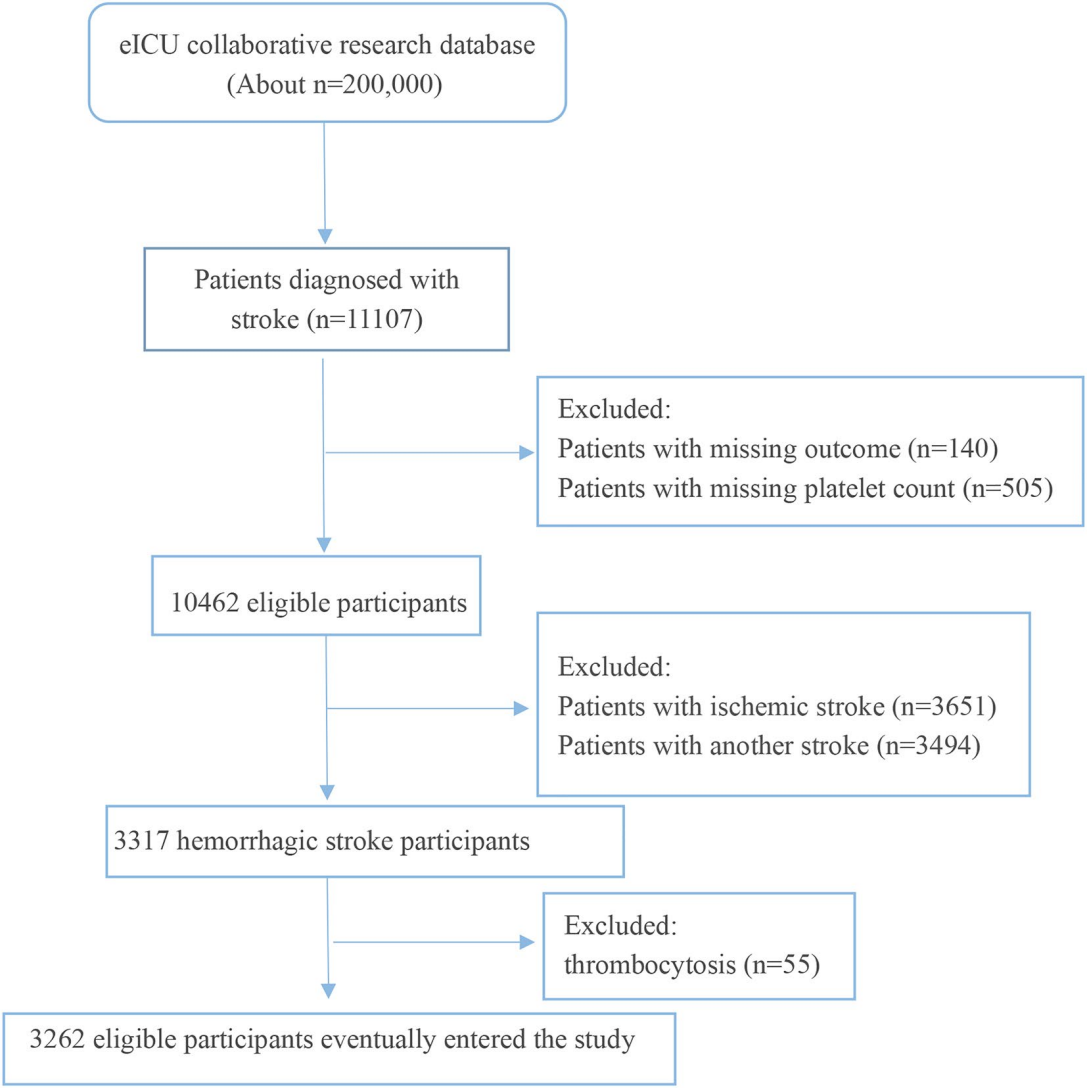
Flowchart of study participants.

This study initially included 11,107 patients diagnosed with stroke. Of them, 140 had missing outcomes, and 505 had missing PC data. Of the 10,462 potentially eligible participants, there were 3651 patients with ischemic stroke, 3494 with other stroke and 3317 diagnosed with HS. After excluding 55 patients with thrombocytosis (PC > 450 × 10^9^ /L)^32^, a total of 3262 patients were included in the study (Fig. 2). The blood platelet parameter at admission was recorded. The primary study outcome was hospital mortality.

### Ethics approval and consent to participate

This study analyzed a publicly available, which permits unrestricted use, distribution, and reproduction. The data was the current secondary analysis, which has not required ethical approval. All methods were carried out in accordance with relevant guidelines and regulations.

## Statistical analysis

The mean and standard deviation was reported for continuous variables with a Gaussian distribution, the median for those with a skewed distribution, and frequency and percentages were used for categorical variables. The Kruskal–Wallis H test (skewed distribution), the one-way ANOVA test (normal distribution), or the χ2 (categorical variables) was assessed to examine the differences between different platelet groups. Following the covariate screening, we built three different models using both univariate and multivariate binary logistic regression models to study the relationship between PC and HS death in hospital. The model was as follows: (i) non-adjusted model (unadjusted covariates); (ii) minimum adjustment model (Model I: adjusted for age, gender, ethnicity); (iii) fully adjusted model (Model II: gender; age; ethnicity; AF, ACS, diabetes, and sepsis). Effect sizes were calculated and reported with 95% confidence intervals (95% CI). Since PC is a continuous variable, the possibility of a non-linear association could not be ruled out. Given the inability of binary logistic regression models in dealing with non-linear associations, we observed an association of PC with the mortality of hospitalized HS patients using the generalized additive model (GAM) as well as smooth curve fitting. The OR values and 95% CI were calculated on both sides of the inflection point. All analyses were conducted using the R statistical software packages (http://www.R-project.org, The R Foundation) and EmpowerStats (http://www.empowerstats.com, X & Y Solutions, Inc, Boston, MA). P-values less than 0.05 (two-sided) were considered statistically significant.

## Data Availability

Data were fully available at https://eicu-crd.mit.edu/.
